# Thematic landscapes and temporal trends of disability technology adoption: insights from Structural Topic Modelling

**DOI:** 10.3389/fdgth.2026.1764516

**Published:** 2026-03-23

**Authors:** M. Kiruthiga, S.N. Vivek Raj

**Affiliations:** VIT Business School, Vellore Institute of Technology, Vellore, India

**Keywords:** artificial intelligence, assistive technology, digital inclusion, disability technology adoption, sign language technology, structural topic modelling (STM)

## Abstract

**Introduction:**

In recent years, the importance of accessible and inclusive technologies has increasingly supported people with disabilities. However, prior studies on the adoption of technology remain fragmented, often focusing on specific disabilities or tools without exploring broader connections. Addressing this, the current study addresses the gaps by identifying core topics, examining temporal variations, and analyzing interrelations across domains.

**Methods:**

By Adapting Structural Topic Modelling (STM), we integrated publication year metadata to uncover thematic shifts in disability technology adoption research. Following the PRISMA framework, 211 English-language articles published between 2015 and 2024 were identified and collected from the Web of Science and SCOPUS databases. Titles, abstracts, and keywords were consolidated, cleaned, and analyzed using the STM in R.

**Results:**

The STM analysis examined six distinct themes: Assistive technologies for a person with visual and physical impairments, m-Health and communication tools for older adults, Robotics integrating cognitive-sensory innovations, Artificial Intelligence (AI)-driven solutions for Cognitive and Sensory challenges, Rehabilitation and Therapy Systems enhancing recovery and mobility, and Educational Technologies with inclusive learning solutions for individuals with disabilities. While six distinct themes were found, and the subsequent topic correlation analysis confirmed their separation, emphasizing negative connections that affirm their conceptual individuality.

**Discussion:**

This study utilizes STM to highlight the growing role of AI in Sign Language interpretation, rehabilitation, and digital learning tools. Beyond technical advances, this study emphasizes inclusive design, policy development, and cross-domain collaboration and areas often overlooked in previous research.

## Introduction

Technology adoption has drastically improved the quality of life of people with disabilities (1) It promotes self-determination, contribution, and social interaction among those with disabilities, and promotes well-being (2). The latest developments in assistive technologies, such as voice-activated home systems and smartphone applications, have significantly assisted individuals with disabilities in implementing daily responsibilities and encouraging independent living (3). Marinaci et al. (4) stated that these innovations promote access to education, employment, and social engagement by lowering physical, cognitive, and participatory challenges.

Assistive tools, such as screen readers and text-to-speech software apps, enhance accessibility in digital environments and facilitate web-based involvement for persons with disabilities (5, 6). Smart home services make daily responsibilities support independent living and promote healthy aging among people with disabilities (7). Even with increasing availability of tech tools, elderly people with vision problems are still experiencing trouble using them because of high costs, inadequate design protocols, and a lack of training for end users, which has resulted in a gap in accessibility (8). Hence, it is essential to incorporate user experiences to ensure that innovation leads to inclusive and right-context solutions (9).

The existing literature consistently frames assistive and digital tech within social, educational, and inclusion-focused frameworks, rather than purely technical paradigms. For example, the study on socially assistive robots highlights the necessity for care technologies to be socially acceptable, ethically sound, and congruent with users’ principles of dignity and trust, therefore promoting technology as a socially integrated system (10). Further strengthening this perspective, studies on therapists’ acceptance of assistive tech demonstrate that academic environments, institutional support, and professional development have a significant impact on adoption, highlighting the educational aspects of inclusion (11).

Research on the use of ICT by elderly people implies that, from a wider societal perspective, ICT adoption promotes societal engagement, reduces isolation, and encourages lifelong learning, all of which strengthen social inclusion frameworks (12). Similarly, studies on older people’s perspectives towards assistive tech enlighten that it promotes autonomy, reduces stigma, and enhances perceived utility, principles closely aligned with participatory and inclusive design principles (13). On similar lines, research on unobtrusive sensing technologies underscores privacy and lack of interference as criteria for social acceptability, demonstrating the importance of inclusion-oriented values in tech design and implementation (14).

To examine the acceptance and utilization of these technologies, research on technology among individuals with disabilities often depends on various frameworks. The Technology Acceptance Model (TAM) underscores perceived ease of use and usefulness (15), and the Unified Theory of Acceptance and Use of Technology (UTAUT) integrates broader factors such as social influence and enabling conditions (16). This research follows the PRISMA framework to identify relevant literature and aggregate the final corpus of articles (17), utilizing Structural Topic Modelling (STM). STM is well-suited for this study as it uncovers hidden topics from large volumes of unstructured textual data and can incorporate metadata to analyze temporal patterns and trends overlooked in previous reviews (18).

Although there has been a rapid expansion of literature on disability technology adoption, the field remains disconnected. The majority of the studies have focused on specific technologies, types of disabilities, or limited thematic areas, such as educational access and workforce engagement (3, 4). Despite systematic reviews and bibliometric analyses contributing to research synthesis, they lack scalable thematic content analysis, dynamic trend tracking, and cross-domain discovery (19). Some reviews have employed thematic synthesis, either manually or through bibliometric mapping (2). Recent studies have leveraged tools such as latent semantic analysis and bibliometric mapping networks (20) to identify research gaps and trends in the literature. However, advanced topic modelling, especially STM, has not been used to comprehensively map thematic developments in this field (18, 21).

This study aims to address the gap in existing research by applying Structural Topic Modelling (STM) to analyse the topics in disability-related technology adoption research and visualize the primary thematic trends of these topics. This study specifically investigated the following research question:

RQ1: What are the main topics explored in disability technology adoption?
RQ2: How are different research topics connected to each other and to various academic fields?
RQ3: Which research domains in disability technology are currently experiencing growth or decline?

## Literature review

Technology implementation is essential for enhancing accessibility for people with disabilities (1, 22). Studies over the past decade investigating the adoption of tech by people with disabilities have closely assessed its effect on public healthcare, education, employment integration, and daily access to tools, outlining its possible benefits and enduring challenges (23, 24). Irrespective of its growth, the field continues to be disconnected, resulting in a gap between technology and impaired communities (4, 25, 26).

Rehabilitative tech addresses both physical and emotional assistance, while telerehabilitation lowers geographical obstacles and improves continuity of care (27). Telecommunication rehabilitation systems, supported by sensors and exergames, are increasingly employed to assess therapy adherence in home-based rehabilitation environments (28). Technologies in the field of communication, such as Augmented Alternative Communication (AAC) devices, voice-output technology, and text-to-speech application software, empower individuals to live independently and encourage valuable social connections (29, 30). Simultaneously, in the education sector, voice-controlled tech, and interactive learning platforms, including ICT-based training and intelligent tutoring platforms, enhance participation for students with impairments. However, their adoption greatly depends on the organizational layout, academic ability, and perceived benefits designed to encourage people with disabilities (2, 31). Technological advances, including mobile applications, smart home automation, and transportation technologies, encourage independent living through by enhanced accessibility.

However, challenges pertaining to digital skills, usability, and accessibility still impact adoption among those with visual impairments (5). Studies on the adoption of new technology in disability typically encompass models such as the Technology Acceptance Model (TAM) (15) and the Unified Theory of Acceptance and Use of Technology (UTAUT) (16). According to research on mobile and digital health tech for older people, these models give attention to perceived outcomes and user experience factors that affect intention to use (32, 33). Despite rising demand and high expenses, progress remains limited due to a lack of training opportunities, and challenges in integrating services and organizations (34–36). The implications of these obstacles are experienced in lower-middle-income countries due to ineffective strategy frameworks and inadequate resources.

Although systematic reviews and bibliometric analyses provide structured results (2), they frequently exclude emerging topics and interdisciplinary connections. Most existing studies concentrate on developed nations and render modest attention to how technology adoption varies across disability types and inclusive design technologies (19). This has led to a fragmented awareness of Inclusive Technology. To address these gaps, STM offers a modular method for outlining key themes, revealing hidden connections, and tracing changes in research over the years (18).

Overall, this study contributes to the disciplines by utilizing a PRISMA-guided corpus and Structural Topic Modelling (STM) to indicate topical structures, trace temporal shifts, and uncover cross-functional interfaces in disability technology adoption. This technique enhances existing reviews by aggregating core adoption theories, such as TAM, UTAUT, and SDT, providing better insights into user acceptance, social influence, and infrastructural elements in assistive and smart technologies. This highlights the demand for long-term research to distinguish innovative strategies and best practices that promote accessibility and upgrade the well-being of individuals with disabilities.

## Methodology

**Figure 1:**
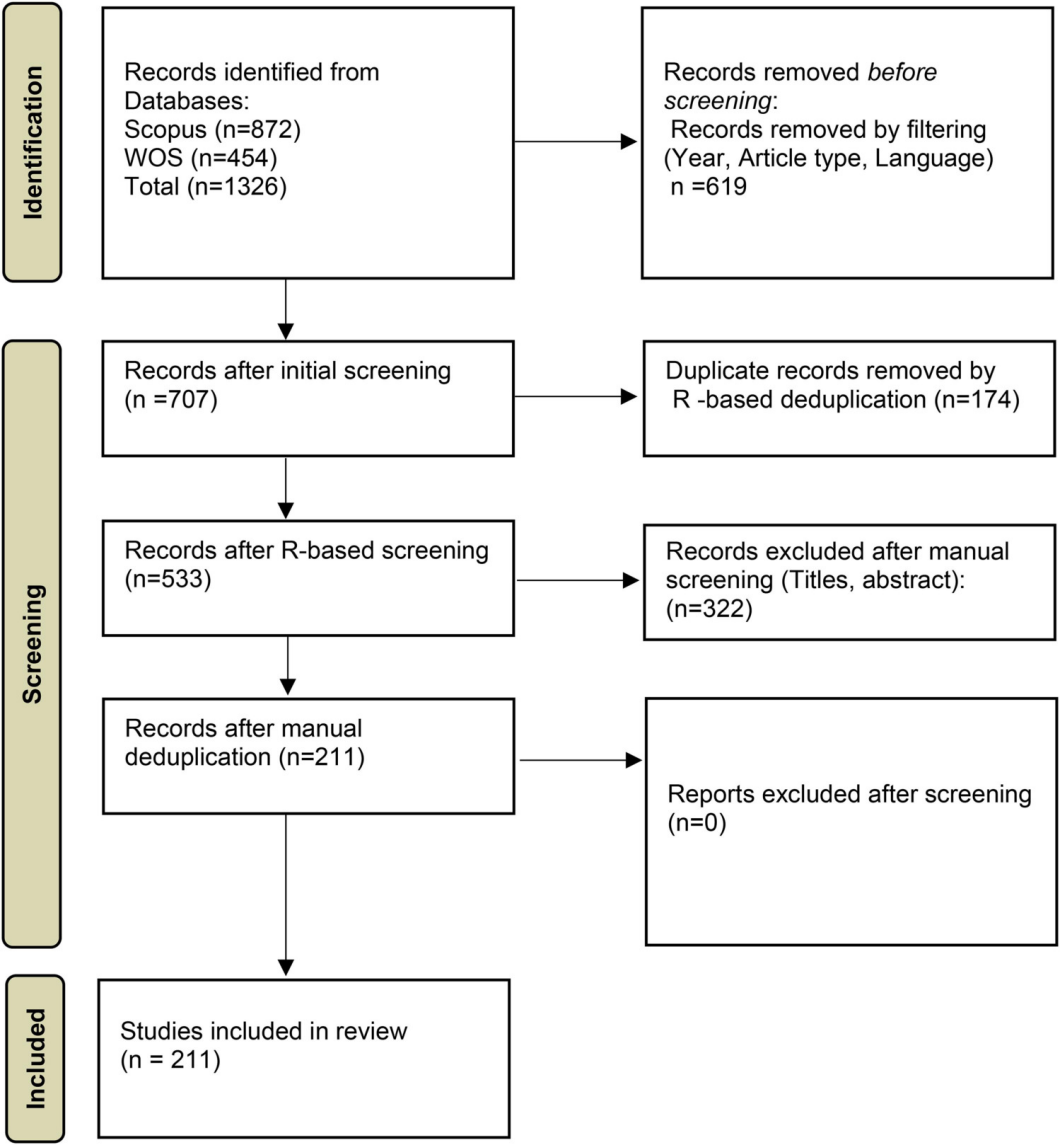
PRISMA. Adapted from “PRISMA 2020 flow diagram template for systematic reviews” by Page et al., licensed under CC BY 4.0.

Figure 1 illustrates the search methodologies performed across Web of Science and Scopus employing different search queries and Boolean variables (“OR” and “AND”) as follows: “technology adoption” OR “technology acceptance” (37) AND “disabled” OR “people with disabilities” OR “special need” OR “Disabled people” OR “accessible” OR “Persons with Disabilities” OR “disability” OR “impairment” OR “PWD” OR “handicap” OR “functional limitation” (38, 39). A total of 454 articles from the Web of Science (WOS) and 872 articles from Scopus were retrieved. Filters were employed in both databases to limit the search to the past decade (2015–2024) of academic publications by centering only on articles. Articles published in English were considered. The data from WOS and Scopus were combined in RStudio using *the Bibliometrix* package, which automatically detected and removed duplicate records based on bibliographic metadata. This process resulted in 174 duplicates, which were removed, and a consolidated dataset with a single file of 533 articles. Titles and abstracts were then manually screened to exclude irrelevant studies, resulting in 211 articles selected for Structural Topic Modelling (STM) analysis.

### Data preprocessing

Data preprocessing involved preparing and cleaning the dataset. Following standard protocols, the titles, abstracts, and keywords of each study were combined to construct the STM dataset (18). To ensure that the data were ready for modelling, the cleanup procedure included removing stop words, non-English characters, numbers, information, and punctuation.

### Structural topic modelling

This study employed Structural Topic Modelling through the stm package in R to reveal hidden themes in a study on how people with special needs adopt technology. STM illustrates a machine learning analysis of massive textual corpora, specifically in social science research (18). The model's implementation of arbitrary metadata enables the assessment of document-level attributes and topic proportions (40). The primary aim of STM is to discover latent topics enclosed between text corpora and estimate their document-specific metadata, thus connecting statistical accuracy to advanced text-mining approaches (40). To measure the optimal number of topics from the STM, the Search K () function was employed, which examines the model fit using held-out likelihood, semantic coherence, exclusivity, and residuals. Based on distinct evaluations, the optimal number of topics was found to be k = 6, which ensured a balance between model transparency and statistical accuracy (40). The year of publication was used as a covariate to determine how themes changed over time. To go deeper into the findings, numerous visualization and diagnostic tools were used. These included estimates of topic quality and trends, topic prevalence plots, word clouds representing core terms, and a topic correlation matrix. The present study also uses natural cubic splines within the STM estimation process to model nonlinear temporal patterns in topic prevalence over three degrees of freedom. The interpretability of topic growth is strengthened by this method, making it possible to estimate temporal relationships across publication years and topic distributions. S. N. Wood (38) found that cubic splines are seamlessly fitted, retrieving both gradual changes and points of divergence in topic prominence while lowering the threat of overfitting. The spline-based modelling technique is well-furnished to mediate an adaptable framework for analyzing topic fluctuation with more elaborate interpretations than linear approaches (18, 41).

## Results

### The main topics explored within the field of disability technology adoption

**Figure 2:**
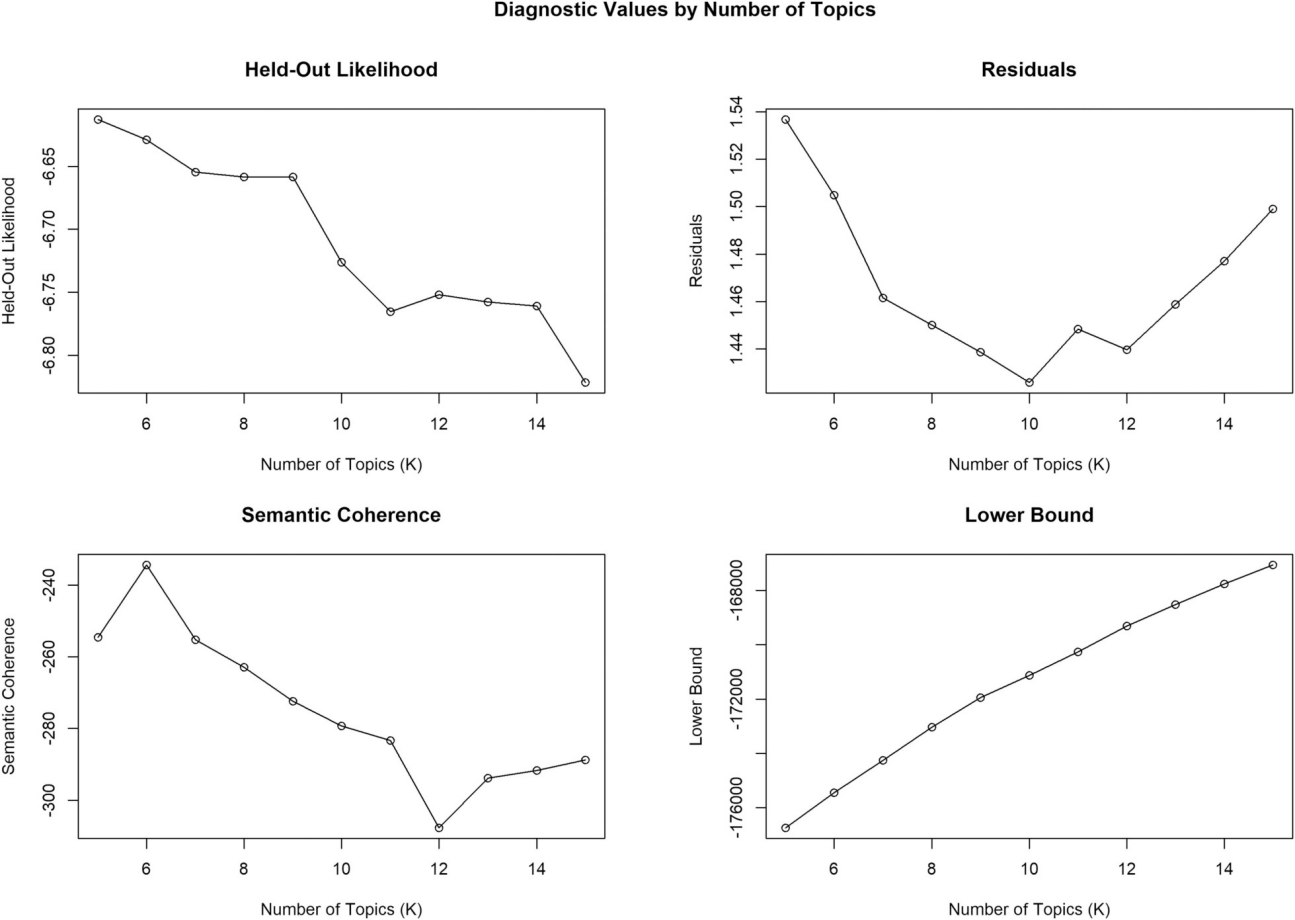
Diagnostic metrics used to determine the optimal number of STM topics, illustrating the model fit and semantic coherence across topic solutions.

The model parameters shown in Figure 2 were applied to determine the optimal values of K. According to semantic coherence, held-out likelihood, and residuals, the preferable result was found at K = 6. This provided an optimal balance between topic interoperability and model accuracy (40). The Held-Out Likelihood parameter is relatively high at K = 6, indicating a better model fit, and then it gradually decreases. Furthermore, semantic coherence peaked at k = 6, signifying that the topics were the most cohesive and interpretable for optimal statistical accuracy and topic reliability regarding disability tech adoption.

**Figure 3:**
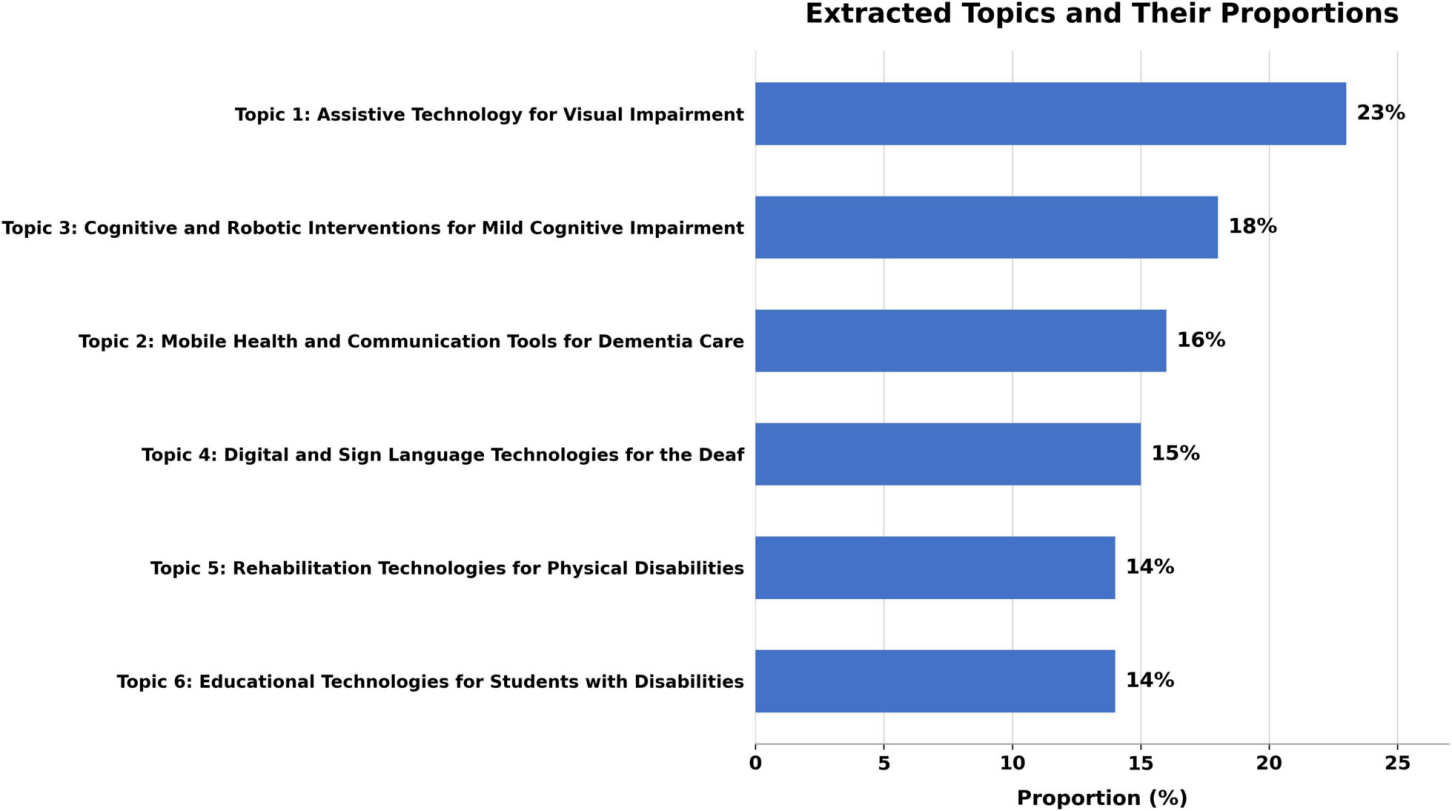
Proportional distribution of STM-identified topics across the analysed literature.

The predominant words for each topic were detected using two methods: highest probability and FREX. These approaches emphasize words that are frequently found and are unique to every topic (18). Table 1 presents the established topic names, their proportions, and prominent articles contributing to every topic. As shown in Figure 3, Topics 1 and 3 revealed higher topic prevalence in the corpus, with 23% and 18%, respectively, followed by Topic 2 (16%), Topic 4 (15%), Topic 5 (14%), and Topic 6 (14%).

**Table 1:** Summary of identified topics from STM.

Topic No	Topic Labels	Topic Proportions	Highest Prob	FREX	Prominent articles
1	Assistive Technology for Visual Impairment. (Visual Assistive Tech)	23%	use, technolog, assist, peopl, older, devic, factor, impair, disabl, accept	visual, vision, product, peopl, ict, impair, influenc, assist, satisfact, affect	[Ahmad et al., (42); Chen, (43); Cho & Lee, (44); Hu et al., (45); Kabir et al., (46); Kan & Wang, (47); Kim, (48); Mak et al., (49); McGrath & Astell, (50); Theodorou, Tsiligkos, et al., (51)]
2	Mobile Health and Communication Tools for Dementia care(M-health)	16%	use, technolog, accept, studi, intent, caregiv, older, particip, communic, applic	communic, dementia, app, mhealth, intent, caregiv, mental, pandem, famili, expect	[De Looff et al., (34, 35); Liu et al., (11); Nogueira-Leite et al., (52); Perez et al., (53); Petrovčič et al., (54); Van Elburg et al., (55, 56); Wang et al., (57))
3	Cognitive and Robotic Interventions for Mild Cognitive Impairment. (Cognitive-Robotic Interventions)	18%	adult, older, cognit, technolog, particip, accept, robot, group, use, assess	robot, sar, cognit, mci, clinician, arm, adult, screen, clinic, engag	(Blanchard et al., (58); Boucher et al., (59); Djabelkhir et al., (60); Isernia et al., (61); JaKa et al., (62); Moore et al., (63); Robinson et al., (64); Rothpletz et al., (65); Seinsche et al., (28)]
4	Digital and Sign Language Technologies. (Sign Language Tech)	15%	technolog, digit, home, peopl, research, health, live, model, use, propos	propos, home, languag, deaf, sign, classifi, implement, digit, print, telecar	[Beckmann, (66); Devlieger, (67); Diraco et al., (14); Groom et al., (68); Jeon et al., (69); Jun, (70); Ortiz-Barrios et al., (71); Ortíz-Barrios et al., (72); Shahin & Watfa, (73); Tielman et al., (74)]
5	Rehabilitation Technologies for Physical Disabilities. (Rehab Tech)	14%	technolog, rehabilit, therapi, caregiv, develop, studi, system, activ, usabl, patient	therapi, fepsim, motor, hand, rehabilit, elder, upper, motion, wrist, challeng	[Babatunde et al., (75); Bianchi, (76); Choi & Maisel, (77); Huang et al., (78); Major et al., (79); Meyer et al., (80); Miclaus et al., (81); Mont et al., (82)]
6	Educational Technologies for Students with Disabilities. (EdTech)	14%	disabl, studi, technolog, use, educ, assist, special, need, factor, research	special, educ, elearn, student, learn, univers, pwds, teacher, financi, children	[Alhwaiti, (83); Bansal et al., (84); Gafoor & Amilan, (85); Huang et al., (86); Ndlovu, (87); Opoku et al., (31); Şahin et al., (88, 120)]

#### Topic 1: assistive technology for visual impairment (visual assistive tech)

Topic 1 examines technologies that assist individuals with visually impairments, emphasizing the functions of ease of use, performance, competitiveness, and equal access in their adoption implementation. These aspects are supported by research adopting the Technology Acceptance Model (TAM) and the Unified Theory of Acceptance and Use of Technology (UTAUT), which emphasize constructs such as perceived usefulness and ease of use as major sources of technology acceptance.

- For example, Ahmad et al. (42) employed TAM to evaluate a grocery shopping assistant app designed for visually challenged users. Their results showed that perceived ease of use was the primary factor that profoundly impacted user acceptance. Customers were positively influenced to adopt the apps because of their simplicity and potential benefits, which enabled them to read product details, such as dietary labels in small fonts, thereby facilitating small-scale shopping.
- Similarly, research utilizing the UTAUT framework (51) indicated that technology adoption among people with visual impairments is influenced by major factors such as performance expectancy. This reflects the effect of mobile-based assistive technologies, such as outdoor recognition tools, indoor guidance systems, and text and object recognition tools, on strengthening productivity.

Prominent articles mapped under this topic highlight the prevalence of utilitarian adoption frameworks in visual assistive tech research. Due to their focus on improving functional performance, efficacy, and autonomy, models such as TAM (42, 45, 46, 49) and UTAUT (51) are particularly adept at explaining user acceptance.

#### Topic 2: mobile health and communication tools for dementia care (m-Health)

Topic 2 focuses on m-Health and communication technologies aimed at encouraging older adults in managing their well-being and communication. Mobile Health apps are increasing in prominence in dementia care, greatly due to their portable design and improved accessibility (57).

- For example (90), investigated interactive television acceptability among elderly people using an interview method guided by TAM constructs, including perceived utility, perceived ease of use, and intention to use.
- Despite this, barriers such as usability issues and high-tech anxiety can be addressed through focused training, outreach programs, and technological literacy programs (13). m-Health and digital tools can strengthen communication, participation, and well-being among older adults, including those with cognitive impairments, specifically when combined with training interfaces (91–93).

The significance of Topic 2 in the STM results illustrates the major studies aligned with this topic use Utilitarian Technology Acceptance Models such as TAM and UTAUT, to elucidate the acceptance of technologies such as m-Health and communication technology by elderly people and caregivers (11, 56). As these technologies are primarily defined by technical support, portability, and productivity in everyday health management and interpersonal relationships, perceived usefulness and ease of use are commonly highlighted as key indicators of user acceptance (11, 56).

#### Topic 3: cognitive and robotic interventions for mild cognitive impairment (cognitive robotics interventions)

Topic 3 studies the interaction of Socially Assistive Robots (SARs), Sensory Technologies, and Cognitive Support Systems in healthcare services and independent living (10). It illuminates the impact of the well-being of older people with disabilities and technological competencies on adoption. It highlights how individual conditions, autonomy needs, and user capabilities shape technology uptake.

- By analysing the top contributing articles in this topic, tech adoption is predominantly examined using utilitarian acceptance frameworks such as TAM, PTAM, and UTAUT (28, 58, 61, 63, 65), especially in studies assessing robot-assisted rehabilitation, digital auditory healthcare, remote rehabilitation systems. These studies commonly associate user acceptance with perceived benefits, ease of use, and user competence.
- Furthermore, research on computerized cognitive stimulation and robot-assisted therapies demonstrates their positive impact on engagement and psychosocial outcomes among older adults and individuals with cognitive impairments (60, 61)

Accessible Technologies, including SARs and virtual cognitive control systems, play an essential improving user involvement (58, 60, 61). The results of these research indicate that technology acceptability is greatly affected by usability, perceived effort, and functional advantages. Furthermore, these technologies improve cognitive, psychosocial, and functional outcomes, thereby fostering the autonomy and well-being of elderly adults with impairments.

#### Topic 4: digital and sign language technologies for deaf (sign language tech)

Topic 4 explored the use of digital empowerment, sign language, and Artificial Intelligence tools to assist people with cognitive and sensory impairments. It targets to improve communication among persons with hearing impairments, including tech advancements such as sign language interpretation and AI-translation (73). Among the top contributing studies within Topic 4, the major focus is on accessibility, equity, and the practical use of technologies in supporting individuals with disabilities, with particular emphasis on inclusive and user-centred approaches (66–68).

The integration of the Ugandan Sign Language transformed communication among deaf individuals in Acholi, facilitating new approaches to social interaction and community development (66). Mobile technologies now assist deaf individuals to engage in social interaction and obtain information independently, encouraging self-empowerment and dignity (94). In addition, Kwak et al. (95) implemented the Technology Acceptance Model (TAM) to assess the ease of use and adoption of the Hearing Rehabilitation for Older Adults (HeRO) mobile healthcare app. Their results demonstrated that factors such as perceived usefulness and ease of use had a substantial impact on senior citizen users’ intention to adopt the app.

#### Topic 5– rehabilitation technologies for physical disabilities (rehab tech)

This topic represents rehabilitation robots (79), therapeutic gadgets (75), and Extended Reality (XR) (96) applications for people with disabilities. The Flexion, Extension, Pronation, and Supination simulator (FEPSIM) is a potential innovative tool revealing demonstrating feasible results for physical disability rehabilitation (97). Nevertheless, elements such as user engagement, system usability, and organisational support affect the sustained adoption of these technologies (80, 82). These studies underscore the significance of practical execution, user-centric design, and application results in influencing technology adoption (75, 80, 82, 97).

- Technology, including the FEPSIM tool and XR-based rehabilitation, has helped individuals recover faster and perform better (79, 81). FEPSIM has been found to be used (98) in therapeutic treatments because of its ease of use and therapeutic value (97).
- However, extended adoption depends on training efficacy, affordability and dependability (76, 82). Inclusive design practices involving patients and healthcare professionals throughout the development process help solve user interface problems and encourage prolonged involvement (80). In addition, practitioners frequently lack the expertise and confidence to engage with m-rehabilitation tools (75). High expenses, broken trust, usability issues often lead to rehab tech abandonment. Park et al. (99) emphasized that stakeholder-inclusive design is emerging as a successful approach.

#### Topic 6—educational technologies for students with disabilities (EdTech)

Topic -6 strongly grounded in formal tech adoption theory, with a significant number of the studies utilizing the TAM and its extensions to explore e-learning, smartphone education and assistive tech usage among people with disabilities (31, 83, 88, 89).

This study analyses educational technology developed for assisting persons with disabilities which promote learning and professional development. Students with physical disabilities need Assistive Educational Tech (EdTech) to succeed and promote independence (31, 87). Information and Communication Technology (ICT) training and E-learning platforms when integrated with accessible technologies such as screen readers, sub headings, and inclusive design are essential in supporting students with disabilities (100). Skill development for career and everyday success requires computer-based training, Information and Communication Technology (ICT) (84).

- For example, research in the United Arab Emirates applied the TAM model to examine the intention of female pre-service special education teachers to implement Assistive Technology (AT) in learning environments. The study examined factors like perceived ease of use, perceived utility, and self-efficacy to train teachers and commitment to assistive technology for encouraging students with disabilities (31).
- Similarly, e-learning adoption in TAM was examined in a research of 1,298 students with special needs, highlighting the importance of constructs such as perceived ease of use, perceived usefulness, and self-determination theory (SDT) constructs such as autonomy, competence, and relatedness. The outcomes demonstrated that most constructs had a significant influence on intention, highlighting the impact of innovation and user experience, along with supportive teachers, in encouraging mobile learning (89).

### Interpretation of word cloud in disability tech adoption

**Figure 4:**
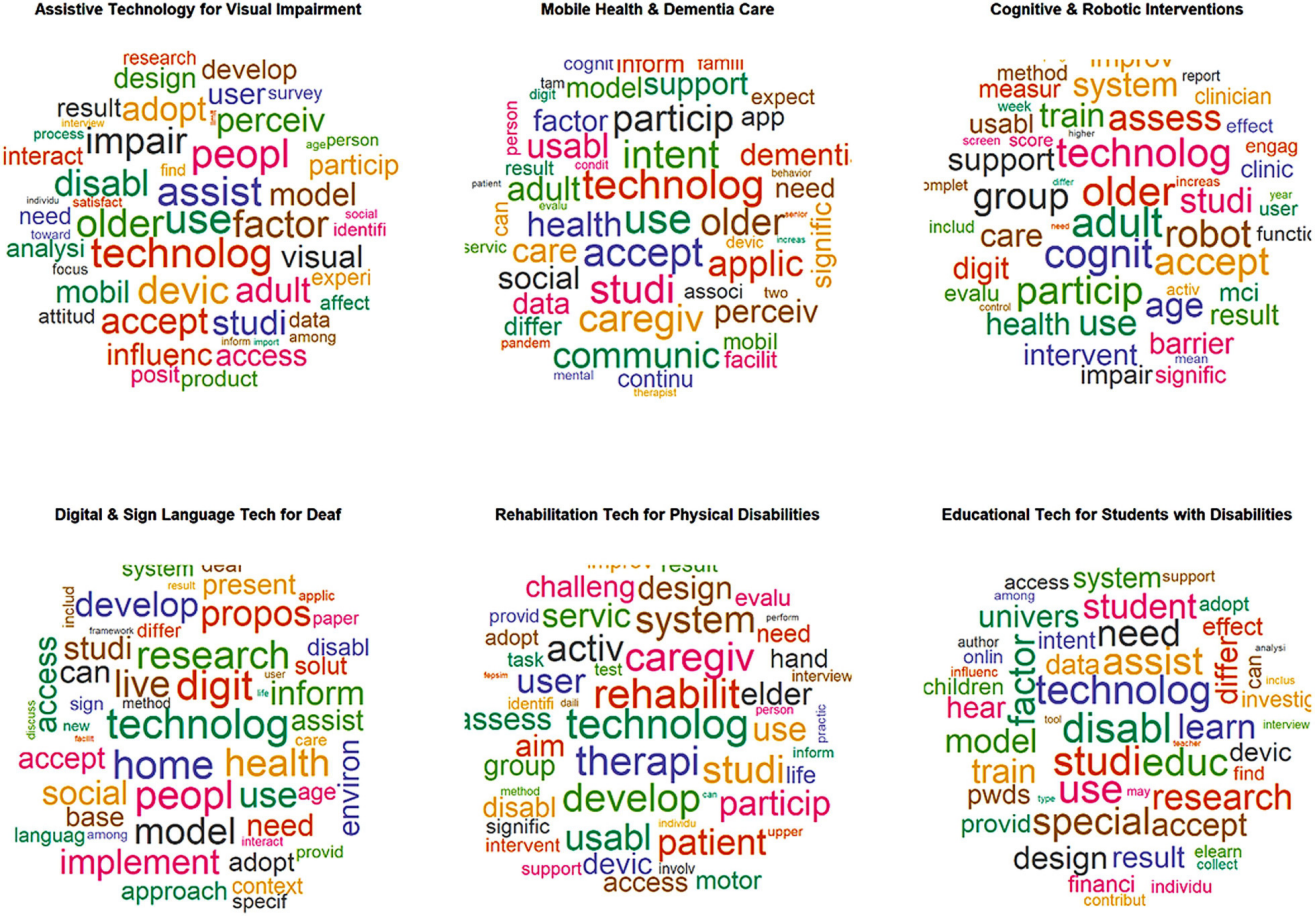
Word cloud visualization of key themes.

The word cloud in Figure 4, derived from the STM, provides a visual representation of six core themes emerging from disability tech studies.

- The thematic focus of Topic 1, Visual Assistive Tech, is characterized by primary key terms such as Visual, Use, Product, Peopl, Access, Impair, and Assist, indicating a strong focus on how assistive technology caters to visually impaired users and the importance of user-centered design.
- The thematic focus on Topic 2-m-Health, highlights the key terms such as Dementia, Communic, Mobile, Health, Use, Technolog, Care, Intent, Caregiv, Usabl, reflecting a growing focus on mHealth interventions for addressing cognitive and functional challenges.
- The central theme of Topic 3-Cognitive Robotics Interventions, focused on key terms such as Robot, Train, Particip, Cognit, Age, Technology, and Care, suggesting that socially assistive robots are increasingly used to support cognitive interventions for mild cognitive impairment.
- The theme of Topic 4**-**Sign Language Tech highlights key terms such as digit, Technolog, Use, Home, Live, Inform, Health, Sign, Language, Access, and Adopt, reflecting advancements in gesture recognition and inclusive communication tools.
- In a similar manner, keywords include Therapi, Motor, Caregiv, User, Servic, and Rehabilit from Topic 5-Rehab Tech indicates sustained growth in therapeutic rehabilitation technics for increasing the strength and improvement.
- Finally, the core themes of Topic 6–EdTech–focus on the primary key terms such as Educ, Learn, Assist, design, PWDs, and train, all of which are interdependent on inclusive edtech and online platforms for specialized education. Overall, the word cloud contributed to promoting and supporting the new thematic construction of tech adoption among individuals with disabilities.

### Assessment of topic quality

**Figure 5:**
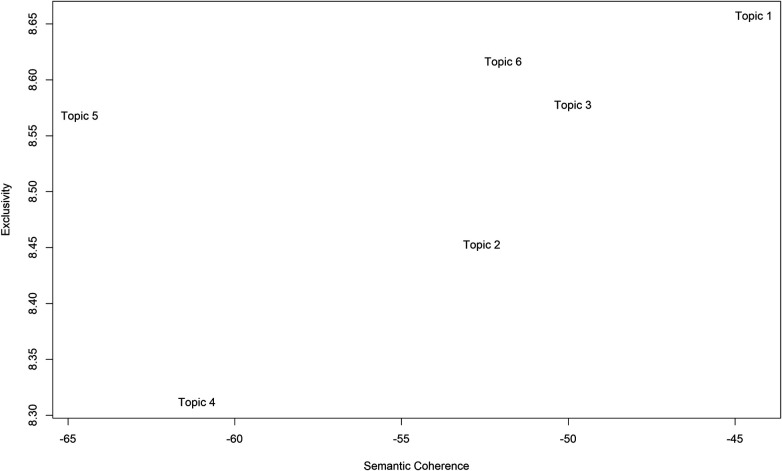
Evaluation of topic quality assessment via semantic coherence -exclusivity analysis.

STM analysis uncovered six latent themes interconnected to disability tech adoption, as shown in Figure 5. Out of these identified topics, Topic 1 -Visual Assistive Tech, has been determined as the most coherent and exclusive topic, signifying its accuracy and specified topic of research. Similarly, Topic 6 -EdTech emerged as a prominent key thematic area, encouraged by its high exclusivity and coherence. Topic 3-Cognitive Robotics Interventions, illustrated a modest degree of coherence and exclusivity, implying that it is growing yet well-established. On the other hand, Topic 5-Rehab Tech exhibited relatively less coherence and moderate exclusivity, signifying unique vocabulary but modest thematic growth. Topic 2- M-Health revealed a relatively low level of exclusivity and partially low-to-moderate coherence, signifying thematic integration and a lack of clarity. Topic 4-Sign Language Tech had lower coherence and exclusiveness, reflecting a relatively lesser thematic coverage. These results bring attention to the STM's role in determining thematic quality, empowering its ability to distinguish well-expressed, coherent, and pervasive themes.

### Different research topics connected to each other and to various academic fields

#### Topic correlation matrix analysis

**Figure 6:**
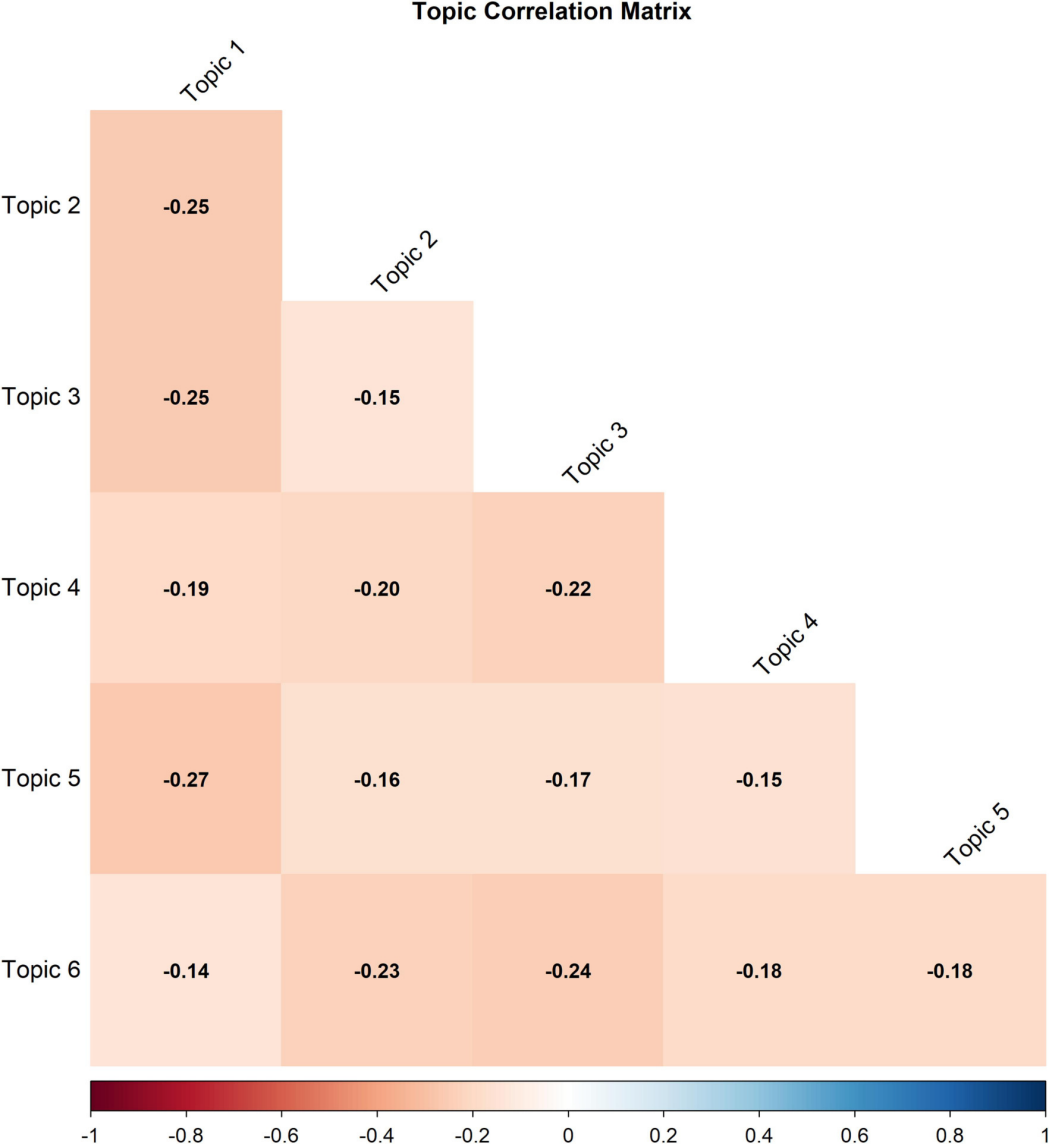
Inter-topic correlation matrix illustrating relationships among STM-identified topics.

As shown in Figure 6, all topics that exhibit correlations are less than zero, signifying negative correlations. For example, the correlation between Topic 1-Visual Assistive Tech and Topic 3-Cognitive Robotics Interventions was −0.25. Likewise, Topic 5-Rehab Tech exhibited a −0.16 correlation along with Topic 2-m-Health.

The negative correlations among the topics suggest that they pay attention to various ideas and do not coincide. As stated by Roberts et al. (18), such correlations usually denote a sharp distinction between topics. This shows that the topics occur in specific areas of discussion and avoid being combined.

These outcomes validate that the STM model efficiently revealed well-articulated and distinct themes related to disability technology adoption. Negative correlations between topics usually suggest little co-occurrence within articles, indicating that themes arise in relatively different discursive domains. This strengthens the strong topic decisiveness and suggests that the STM model has successfully extracted mutually exclusive and well-separated themes across disability technology adoption.

### Research domains in disability technology are trending increasing and decreasing

#### Spline -based topic trend analysis

RQ3 aims to illuminate the temporal evolution of research on technology adoption among persons with disabilities by identifying both rising and declining areas of inquiry in the literature. Topics that show increasing thematic interest may gain academic significance, while those with consistently declining themes may gradually diminish in academic relevance.

**Figure 7:**
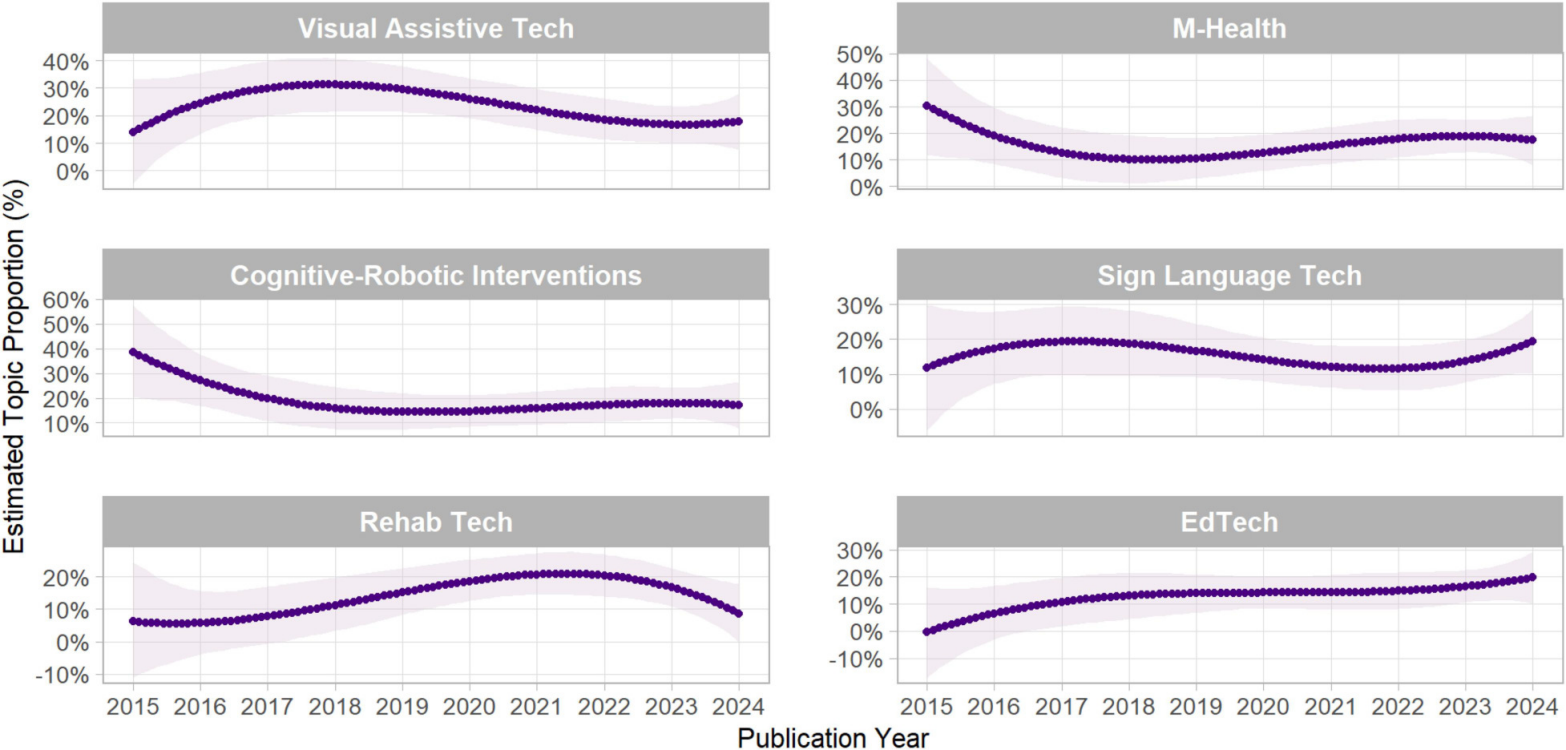
Temporal dynamics of STM-identified topics over time.

- Figure 7 shows that the popularity of these topics changed noticeably over time. As indicated, Topic 1-Visual Assistive Tech, showed increased prominence from 2015 to 2018 has steadily decreased since 2018 and eventually stabilized after 2021.
- The trend for Topic 2, M-Health, reflects a fluctuating landscape, with a decline from 2015 to 2018, a rebound through 2022, followed by a slight mild downturn.
- Topic3 -Cognitive Robotics Interventions gradually declined until 2019, and the trend stabilized from 2021.
- Topic 4—Sign Language Tech steadily increased between 2015 and 2017, declined from 2018 to 2021, and showed a sharp revival from 2021 to 2024.
- Topic 5 (Rehab Tech) experienced a shift in focus after 2021, with academic attention moving toward the specific applications of cutting-edge technology. Finally, Topic 6 (EdTech) demonstrated steady growth after 2020.

## Discussion

This study aims to deliver a systematic investigation of disability tech adoption research by incorporating PRISMA-directed corpora and structural topic modelling (STM). The study revealed six thematically unique topics, each of which represents a critical aspect of the discussions regarding technology adoption in the framework of disabilities. These themes are appropriate for various disability organizations and user engagement and embrace a wide range of technological innovations. Topic 1 examines Visual Assistive Technologies, and some of the prominent research in this area has been conducted using Technology Acceptance Model (TAM) and the Unified Theory of Acceptance and Use of Technology (UTAUT). These research emphasizes the significance of core constructs, including perceived usefulness, ease of use, performance expectancy, and effort expectancy, in the adoption of tech by visually challenged individuals (42, 51). However, the studies also suggest that adaptive technology use by individuals with visual impairment frequently indicates distinctive constraints, including large expenses and financial difficulties, which continue to influence the diffusion of assistive tech use by people with visual impairment (101).

Topic 2 concentrates on the persisting challenges that continued to shape m-health participation, where accessibility issues are still common, particularly among elderly persons with cognitive impairments. Research suggests that programs for digital literacy, guided support, and structured training can improve individuals’ confidence, involvement, and well -being while utilizing these technologies (13, 91–93).

Topic 3 indicates work in which adoption of robotic and cognitive assistive technologies is frequently evaluated through UTAUT oriented perspectives, especially involving user perception, ease of use, and system adaptability (28). Recent research (2020–24) indicates a shift from experimental prototypes to integration in real settings, increased implementation issues, and cross-disciplinary development themes (102–104).

Although the majority of Topic 4 deals with sign language and Deaf communication technology, parallel breakthroughs in hearing rehabilitation, such as the HeRO app examined by TAM, indicate complementary usability -driven acceptance techniques (95). The growth in this research topic observed post-2021 likely reflects advancements in AI-driven accuracy and deep learning architectures, which have significantly enhanced translation accuracy and communication accessibility for the deaf community (105).

Similarly, Topic 5 addresses rehabilitation technologies, where studies such as (97) tend to prioritize practical implementation issues, usability, and practicality rather than traditional behavioural acceptance frameworks. Finally, Topic 6 centred on EdTech, revealing a growing body of literature in which some prominent studies utilize TAM and SDT to address adoption barriers and provides a foundation for enhancing user perceptions of utility, usability, autonomy and competence (89). Research underscores the collaborative impact of enhancement of skills, institutional support and tech-mediated training in enhancing participation for individuals with disabilities (84). Moreover, in post-2020, the expansion of EdTech focused on disabilities has been driven by the rapid digitization prompted by COVID, the enduring nature of hybrid learning models, and advancements in AI that have integrated inclusive tools into mainstream education (106, 107).

Taken together, the analysis of key representative studies within each topic illustrates a clear match between the type of disability tech examined and the theoretical frameworks used. The dominance of utilitarian adoption models like the Technology Acceptance Model (TAM) (12, 42, 46, 108) and the Unified Theory of Acceptance and Use of Technology (UTAUT) (51, 109, 121), which indicates a strong emphasis on functional efficiency, usability, and performance -oriented outcomes, is consistent with the dominance of visual Assistive tech (Topic 1) and m-Health solutions (Topic 2). This is because these models are especially well-suited to technologies designed to facilitate task completion, autonomy, and productivity. Consequently, they are broadly used in the fields of vision and health (49, 90).

However, topics that prioritize inclusive design, accessibility, and real-world implementation (Topics 4 and 5) demonstrate a diminished dependence on intention-based acceptance models, instead reflecting user-driven and practice-oriented perspectives (9, 90, 97, 110). This shift implies that traditional acceptance frameworks may be inadequate to account for contextual, social, and institutional influences on adoption as disability technologies mature and transition to sustained everyday use (2, 13). Meanwhile, Topic 6 examines on motivational explanations of technology adoption, especially through Self-Determination Theory, where autonomy, competence, and related ness strongly influence involvement in inclusive digital learning (89).

The studies reinstate that TAM and UTAUT are still essential for comprehending early-stage and function-driven adoption, yet the future disability technology research would be enhanced by broader socio-technical and inclusion-oriented theoretical approaches that more comprehensively consider lived experiences, service ecosystems, and institutional environments (10, 12).

The systematic barriers related to cost, infrastructure, and policy are further reinforced by evidence on assistive tech distribution, which reveals disparities, as users from lower socio economic communities have difficulties in finding and maintaining access to technological aids (78, 87). The correlation matrix shows that certain topics are conceptually distinct, with negative correlations of −0.27 between Topics 2-M health and Dementia and Topic 5-Rehab Tech and −0.16 between Topic 1 -Visual Assistive Tech and Topic 3-Cognitive Robotics. According to (40), negative correlations can be taken as showing conceptual separation between thematic domains. This insight is beneficial for future efforts to address the gaps in several fields, including education, robotics, healthcare, and policy guidelines.

By incorporating STM, this review offers an elaborate and adaptable framework for the integration of technologies embraced in the field of disability domain. It expands further standard evaluations by revealing new areas like AI-powered sign language software for recovering and learning management systems, which acquired limited attention in past research. The study indicates that TAM and UTAUT have a significant role in analysing technology adoption (42, 51, 90). Meanwhile, the use of SDT explains how persuasive factors influence users intentions and involvement with cognitive and intelligent systems (89). This review improves to academic discourse by examining significant social challenging, demonstrating how cutting-edge technologies can foster inclusivity, examine adoption challenges, and empower societies over design thinking.

## Practical implications

### Technology development and service delivery visual assistive technology

The establishment of six thematic areas promotes inclusivity and extends usability testing a broader variety of technologies. Topic proportions show that Topic 1, Visual Assistive Technology (23%), remains the most broadly researched area. However, the downward trend in research output in recent years can be attributed to various features, including technological development, a shift in the priorities of future research against Artificial Intelligence and Data Analytics, Design and Financial challenges, and COVID-19 impacts.

However, despite this downturn, several sectors like AI enabled computer vision, navigation and mobility assistance, wearable assistive tools and smart-phone based visual support systems still remain innovative (111–113). This changeover suggests the need for innovative transformation through multidisciplinary integration, user-centric AI implementation, data-driven applications, and decision support that encourages the digitization of current technologies rather than individual breakthroughs.

### Integrated cognitive, robotic, and assistive system design: cognitive -robotic interventions

Furthermore, the topic correlations reveals disconnections among Topic 1 -Visual Assistive Tech and Topic 3-Cognitive Robotics, highlighting persistent fragmentation and limited cross-domain integration (20, 114). To address these gaps, it is essential to develop cross-disciplinary monetary initiatives designed to support integrated robotics, cognitive science, assistive tech projects (72, 80). Such initiatives should foster the development of hybrid technologies that integrate Assistive, Rehabilitative, and AI-driven features to enhance the user experience (47, 74). Meanwhile, continuous collaboration among tech developers, healthcare professionals, and educators to collaboratively develop inclusive solutions (11, 87).

### Rehabilitation and community practice: rehabilitation technologies

Key articles charted under Topic 1 and 3 reveal that the adoption of smartphone navigation aids, wearable vision sensors, AI-driven vision technologies, and robot assisted rehabilitation systems is impacted more by workflow integration, therapist guidance, and cost -effectiveness than by technical innovation highlighting the importance for interoperable systems and ongoing professional support in rehabilitation and community services (11, 28, 115, 116).

### Accessible communication and sign-language technologies

Despite increased scholarly attention, enduring barriers associated with cost, interoperability, and equitable access persist, highlighting the importance of greater user participation in technology design and the development of equitable implementation frameworks (117). To address this, future research should focus on developing cross-compatible accessible systems, systematic datasets, and performance benchmarking to facilitate the practical implementation of hearing-impaired communities (29, 30). Furthermore, promoting experimental pilot programs could encourage the practical evaluation and development of AI-driven sign language technologies and mobile communication tools, facilitating their transition from experimental design to feasible solutions.

### Education and digital inclusion

The steady growth of Topic 6 (14%), educational and digital inclusion technologies post-2020, emphasizes the constant and increase in the demand for learning management systems (LMS) platforms that promote numerous formats to access content such as text, audio, captions, and digital sign overlays (118, 119). According to these findings, the adoption of technology in education for students with disabilities implies not only easily accessible platforms but also constant institutional investment in inclusive digital governance frameworks, technical support, and teacher training (84, 89).

To promote inclusive education, organizations should concentrate on implementing assistive tech tools into teacher preparation programs, since further training can help minimize opposition and enhance inclusive teaching methods (31, 87). Educational Institutions should also Promote the acceptance of multi-format educational resources in schools and colleges to better accommodate students with different impairments. In addition training sessions for educators and as well as administrators can be administered to promote successful deployment of Learning Management Systems (LMS) and assistive tech (83).

### Policy and governance and public deployment implications

Overall, the findings emphasize the responsibility of policymakers and regulators to support governance structures and promote coordinated, equity-driven policies in order to support the long term development and implementation of inclusive technologies (82, 116). In addition, standardize accessibility policies for educational tech (87), rehabilitation, assistive tech across institutions (47), promote equitable allocation of resources for low-and middle -income environments including infrastructure, gadgets, and internet access (68).

Key Studies on AI-driven sign recognition, translation systems, and smartphone communication technologies reveal methodological progress while simultaneously highlighting significant variability in datasets, evaluation protocols, and operational contexts. These conditions indicate that translation from laboratory innovation to scalable public deployment remains challenging, underscoring the importance of coordinated pilot implementations, shared data infrastructures, and benchmarking practices (29, 30).

However, instead of supporting distinct tech innovations, cross-disciplinary funding calls that integrate robotics, AI, rehabilitation, and assistive tech research are necessary as indicated by the observed thematic disconnect between major topics (20, 114).

### Limitations

This study applies Structural Topic Modelling (STM) to examine the scalable analysis of research dynamics in disability technology adoption to validate the use of multi-framework interpretations and trend-based investigations. First, limiting the analysis to abstract-level data reduces the ability to explore detailed discussions in full texts, however, eliminate methodological complexities and detailed discussions. Some elaborate conceptual relationships or integrated subthemes may be inaccurately portrayed Second outcomes vary with the number of topics selected, though Six topics were chosen through statistical metrics and qualitative expert evaluation. Alternative topic numbers could identify alternative subthemes or enhance existing ones. Third, the inclusion of English excludes research from non-English-speaking regions especially in the Global South limiting adaptability. Finally, it is important to acknowledge that the STM, incorporating spline-based trend analysis, was applied to a deliberately curated corpus of 211 articles aligned with the research objectives; therefore, the observed topic prevalence and temporal trends should be interpreted as descriptive and exploratory patterns within the analyzed literature rather than statistically generalizable findings.

### Future research agenda

STM enables the identification of significant topics and trends without any subjective bias. In future research, bibliometric approaches may be employed to map publication patterns, collaborative authorship, and citation influence, and a Systematic Literature Review (SLR) would provide a structured framework for future work, accessing scholarly dynamics, and integrating existing findings. Beyond bibliometric and SLR, future research can be strengthened by including meta-analysis and integrative reviews. A meta-analysis statistically connects results from numerous sources, procuring a more detailed and contingent estimation of effects, remarkably adjusted with the thematic cluster exhibited by the STM.

## Conclusion

This study revealed PRISMA and Structural Topic Modelling (STM) to analyse how research techniques on disability technology adoption is systemized. By reviewing 211 articles, the study indicated six core themes: Assistive Technology for Visual Impairment (Visual Assistive Tech), Mobile Health and Communication Tools for Dementia care (M-Health), Cognitive and Robotic Interventions for Mild Cognitive Impairment (Cognitive Robotics Interventions), Digital and Sign Language Technologies for Deaf (Sign Language Tech), Rehabilitation Technologies for Physical Disabilities (Rehab Tech), and Educational Technologies for Students with Disabilities (EdTech).

Current trend patterns reveal that EdTech, and sign language technology have demonstrated increasing attention, while mHealth and visual-assistive technology have acquired less attention, indicating the demand for new approaches. The trend in Cognitive Robotic Intervention shows a steady but moderate academic interest in AI-Powered robotic intelligence to improving cognitive abilities and rehabilitation care. The correlation matrix states low negative correlation between themes, signifying that healthcare services, education systems, and user interface domains emerge as distinctive themes in the literature. Although these fields are thematically distinct, their similarities demonstrate the importance of future interdisciplinary research.

This study's contribution is its use of systematic review and computational text analysis to create a theoretically sound and scalable way to map the literature. The findings reveal the importance of inclusive design, user-centric design, domain integration, and multilingual research to bridge accessibility gaps. Strengthening collaboration among developers, policymakers, and researchers is essential for building adaptive, equitable, and future-ready assistive ecosystems.

## Data Availability

The data analyzed in this study is subject to the following licenses/restrictions: Data used in the research is sourced from Scopus and Web of Science Databases using the search terms provided in the article text. Requests to access these datasets should be directed to vivekraj.s@vit.ac.in.
